# The role of nurse-client relationships in maternal and child healthcare: a qualitative study in rural Tanzania

**DOI:** 10.3389/frhs.2023.1058840

**Published:** 2023-06-26

**Authors:** Kahabi Isangula, Loveluck Mwasha, Eunice Pallangyo, Eunice Ndirangu-Mugo

**Affiliations:** ^1^ School of Nursing and Midwifery, The Aga Khan University, Dar Es Salaam, Tanzania; ^2^ School of Nursing and Midwifery, The Aga Khan University, Nairobi, Kenya

**Keywords:** provider-client interaction, provider-client relationships, nurse-client relationship, benefits, maternal and child care, rural, Tanzania, Africa

## Abstract

**Background:**

The literature suggests that poor provider-client relationships in maternal and child healthcare (MCH) continue to impact healthcare service uptake, continuity of care, and MCH outcomes. However, there is a paucity of literature on the benefits of the nurse-client relationship for clients, nurses, and the health system, particularly in rural African contexts.

**Objective:**

This study examined the perceived benefits and disadvantages of good and poor nurse-client relationships in rural Tanzania respectively. We present the findings of a community-driven inquiry that was the first step of a broader study that sought to co-design an intervention package for strengthening nurse-client relationships in MCH in rural contexts using a human-centred design approach.

**Methods:**

This study used a qualitative descriptive design. Nine focus group discussions and 12 key informant interviews were conducted using semi-structured interview guides. Participants were purposefully selected nurses/midwives and clients attending MCH services, and MCH administrators. Data were managed using NVivo and analysed thematically.

**Results:**

A range of perceived benefits of good nurse-client relationships and disadvantages of poor relationships emerged. Perceived benefits of good nurse-client relationships included: (i) benefits to clients (increased healthcare-seeking behaviours, disclosure, adherence, return to care, positive health outcomes, and referral tendencies); (ii) benefits to nurses (increased confidence, efficiency, productivity, job satisfaction, trust, and community reputation and support); and (iii) benefits to healthcare facilities/systems (increased client load and consequently income, fewer complaints and legal disputes, increased trust and facility delivery, and reduced maternal and child deaths). The disadvantages of poor nurse-client relationships were basically the opposite of their benefits.

**Conclusion:**

The benefits of good nurse-client relationships and the disadvantages of poor relationships extend beyond patients and nurses to the healthcare system/facility level. Therefore, identifying and implementing feasible and acceptable interventions for nurses and clients could pave the way for good nurse-client relationships, leading to improved MCH outcomes and performance indicators.

## 1. Introduction

Nurses and midwives continue to be essential in promoting population health and well-being by providing psychosocial and medical care in various settings and acting as client advocates (1–3). Furthermore, worldwide, nurses and midwives constitute a large percentage of the healthcare workforce that is charged with providing maternal and child healthcare (MCH) services (1, 2). In sub-Saharan Africa, nurses and midwives offer healthcare services and education on the diverse healthcare needs of the mother, newborn, and children, thereby positively influencing the achievement of desired health outcomes (1). Evidence has shown that an adequate and well-supported nursing and midwifery workforce could avert up to 83% of maternal deaths, neonatal deaths, and stillbirths (4). Moreover, competent, responsive, and compassionate nurses and midwives are critical for enhancing client uptake and continuity of care, along with the quality of services, positive health outcomes, and practices. For example, they contribute to strengthening breastfeeding practice and reducing unnecessary cesarean sections, maternal infections, postpartum hemorrhage, and preterm births (5). Therefore, investing in nurses and midwives can yield enormous (i.e., 1,600% or 16-fold) gains because of improved MCH outcomes (6).

At the heart of nurses' and midwives' critical role in healthcare is the value of strong nurse-client relationships. Evidence from the literature equates good nurse-client relationships to improved quality of care, openness, respect, and cost-effective care (7–9). Conversely, poor nurse-client relationships are associated with low use of facility-based care, dissatisfaction with care, default from follow-ups, and lack of trust in healthcare providers (10–13). There is a large body of literature on nurse-patient relationships. Some studies have focused on quality care and asserted that good nurse-client relationships contribute to improved quality of care and patient satisfaction (10, 14–16). Other studies focused on client involvement or collaborative care where the patient and family participate in planning care and decision-making (6, 10, 14, 17), or on communication about care received, including diagnostic tests (4, 5, 12, 18). However, few studies have specifically focused on the benefits of the nurse-client relationship for clients, nurses, and the healthcare system, particularly in rural African contexts. To partly address this gap, this paper examined the perceived benefits of good nurse-client relationships and disadvantages of poor nurse-client relationships in rural Tanzania. A focus was to identify the perceived impacts of nurse-client relationships on the clients, nursing staff, and the healthcare system. We present the findings of a “community-driven inquiry” which was the first step in a broader study focused on co-designing an intervention package to strengthen nurse-client relationships in MCH in rural contexts using a human-centered design (HCD) approach (19). The results are expected to cement the value of strengthening nurse-client relationships as an entry point for improving client uptake, continuity of care, and health outcomes.

## 2. Methods

### 2.1. Design

The protocol for the broader HCD study has been published elsewhere (19). In summary, a five-step HCD approach was employed as a framework for co-designing of an intervention package for and strengthening the nurse-client relationships embracing qualitative descriptive design using focus group discussions (FGDs), key informant interviews (KIIs), and consultative meetings. This study was the first step of the HCD study which sought to explore both the benefits and disadvantages of nurse-client relationships and the contributors to poor nurse-client relationships. Qualitative descriptive design was regarded more appropriate for this inquiry in answering two key questions: (i) What are the benefits of nurse-client relationships in MCH care in rural Tanzania? and (ii) What are the disadvantages of poor nurse-client relationships in MCH care in rural Tanzania? Furthermore, a qualitative descriptive approach is more appropriate for this study as it was aimed at generating a rich understanding and describing the benefits and disadvantages of nurse-client relationships without testing an existing theory (19–21). HCD is an approach to solving complex problems by using a series of iterative and habitually nonlinear steps to develop solutions (19). In the HCD approach, beneficiaries, or end users are invited to partner with the research team in exploring the challenges, designing and evaluating the emerging solutions to better comprehend, and solve the challenges they have identified. A mixed method design was considered but deemed impractical for this inquiry because we focused on generating rich perspectives on nurse-client relationships to guide further HCD steps.

### 2.2. Settings

This study was conducted in Shinyanga, a region located in Tanzania's Lake Zone and predominantly inhabited by Bantus. A previous study (11) provided a detailed description of the region. Briefly, Shinyanga is a low-income region that is administratively divided into five districts: Shinyanga MC, Shinyanga DC, Kishapu DC, Kahama MC, and Kahama DC. Shinyanga was chosen for this study for two reasons. First, ethnically the region is predominantly inhabited by Sukuma who share a range of socio-cultural beliefs and practices with minimal diversity; this near homogeneity meant the region offered a perfect example of many rural regions of Tanzania. Second, despite several capacity-building interventions, local data indicated there were major concerns about poor nurse-client relationships in MCH (11). From Shinyanga region, Shinyanga MC was purposefully selected because patients in these districts have good access to the formal healthcare system (primarily public and few private and faith-based facilities) and traditional care (11).

### 2.3. Study population, sample size, and sampling method

Nine focus group discussions (FGDs) were conducted with purposefully selected nurses and MCH clients. We conducted four FGDs with 30 nurses (26 female and 4 male) and five FGDs with 36 MCH clients all females. Each FGD involved 6–8 participants and nurses and clients were interviewed separately. This was done purposefuly to minimise the potential power dynamics between nurses and MCH clients. We also conducted 12 key informant interviews (KIIs) with MCH stakeholders. Although equal representation is not a primary focus in qualitative studies (19–21), the level (dispensary, health center, and district hospital) and ownership of the facility (public, private, and faith-based) were considered during participant enrollment. No requirements around participant age or other characteristics were set for this qualitative inquiry, but we included: nurses that had worked in MCH for at least 2 years; clients that had attended at least three MCH clinic visits in a year; and MCH service administrators in Shinyanga. No participants dropped out of the interviews because adequate information was provided prior to participants' enrollment.

### 2.4. Data collection tools

Semi-structured guides were developed for the FGDs and KIIs. These guides were translated from English to Swahili through a consultative process involving experts at the Aga Khan University. First, the English versions of the guides were translated into Swahili, and then back translated to English and checked for conceptual equivalence. Questions in the interview guides (Supplementary Material S1) examined participants' experiences of poor and good nurse-client relationships, factors contributing to poor nurse-client relationships, and suggestions and considerations for improving nurse-client relationships. The “poor relationship” was conceived as any therapeutic encounter between a nurse and a client that was characterized by negative experiences that may have included (but not limited to) the nurse's expression of disrespect for the client's views and opinions, lack of sense of trust, lack of honesty and transparency, lack of sense of commitment or care, criticism, sarcasm and/or abuse, lack of listening skills and understanding clients' body language etc. The “good relationship” was conceived as any therapeutic encounter between a nurse and a client that was characterized by positive experiences that may have included (but not limited to) the nurses' expression of respect for patient views and opinions, sense of trust, honesty and transparency, sense of commitment or care, good listening skills and understanding clients’ body language etc. Most of these have been documented previously as factors shaping good and poor provider-client relationships in a similar setting (10, 21). Prompts in the interview guide included benefits of good and disadvantages of poor nurse client relationships to the nurses, clients and health system.

Three research assistants including two female and one male, with diplomas in health sciences and had previously worked in various health-related projects and research. They were recruited and trained on using interview guides and techniques for this study. The interview guides were pre-tested in purposefully selected settings, which were later excluded from this study. After pre-testing, the guides were refined to ensure readiness for use in the actual data collection process. Close and supportive supervision of the research assistants was ensured throughout the data collection and analysis stages to safeguard data quality.

### 2.5. Participant recruitment

Recruitment for FGDs commenced through purposeful selection of healthcare facilities based on ownership, level, and availability of MCH services. A courtesy visit was made to the Shinyanga District Medical Officer to secure approval to visit the facilities. Next, physical introductory visits to the facilities were conducted, and study information was provided to the facility in-charges, who assisted with identifying enrolment assistants. The enrolment assistants were briefly oriented on the study, and then they shared information about this study during clinical meetings and MCH visits and registered those who expressed interest. This was followed by subsequent visits to the facilities by research assistants to schedule and conduct FGDs. Recruitment for the KIIs was completed by initial phone contact with MCH administrators after obtaining their phone numbers from the district medical office. During these phone calls, interviews were scheduled with consideration of participants' preferences for place and date.

### 2.6. Conducting interviews

The interviews were conducted at places and dates preferred by the participants. Before the FGDs and KIIs started, participants were given information about this study and risks and benefits of participation (an information sheet was part of the interview). Verbal consent for the interview and voice recording was sought in advance and recorded as part of the interview. The interview sessions lasted approximately 30–60 min and were held in safe environments. Data saturation was achieved after conducting 9 FGDs and 12 KIIs where conducting subsequent interviews was deemed unnecessary. Research assistants maintained filed notes, the contents of which facilitated the identification of challenges during data collection and generating collective solutions and guided the development of the project reports, the methods section of this publication, and the reflective analysis of the results. Because of the recent COVID-19 pandemic, all participants and research assistants were provided with face masks and hand sanitizer. Social distancing was maintained throughout the FGDs and interviews.

### 2.7. Data management and analysis

Because of the research objectives, the qualitative data were analysed using the thematic analysis strategy as described by Braun and Clarke (20). A stepwise approach was used for a deductive thematic analysis of the interview transcripts. First, the research team examined the research questions and interview guide and generated several themes and subthemes on a consensual basis. This resulted in an analytical matrix of the main themes and subthemes. Next, the transcripts of the KIIs and FGDs were reviewed and phrases (codes) representing participants' responses to the investigators' probes were exported to relevant themes and related subthemes in NVivo version 12 (QSR International). The research assistants conducted coding under close supervision by the principal investigator. The research team then used a consensus-based approach to determine whether to include codes that did not fit within the developed subthemes and themes and discard those that were subjectively and objectively deemed of no critical value to this study. Then, the data from NVivo were exported to MS Word for report generation.

## 3. Results

### 3.1. Participants' demographics

This study involved 12 KIIs with MCH administrators and stakeholders and nine FGDs with nurses and MCH clients. The majority of KII participants were female (67%), aged 40–49 years (67%), had a university education (75%), and had more than 5 years of experience in MCH leadership (75%). The majority of nurses who participated in FGDs were female (87%), aged 30–39 (46%) with college and university education (77%).All MCH clients during FGDs were female partly pointing to female gender dominance in MCH service utilization in this rural region. In addition, majority of MCH clients were aged less than 30 years (61%) with no or primary education (61%). (Table 1). The education disparity between nurses and clients may create power dynamics that may negatively impact interpersonal relationships (23, 24).

**Table 1:** Participant demographics.

	KII PARTICIPANTS (*N* = 12)	FGD PARTICIPANTS (*N* = 66; 5 FGDs for clients and 4 FGDs for nurses)	
	MCH STAKEHOLDERS	CLIENTS	NURSES
	Number (%)	Number (%)	Number (%)
Gender			
Male	4 (33)	0	4 (13)
Female	8 (67)	36 (100)	26 (87)
Age			
<30	0	22 (61)	6 (20)
30–39	3 (25)	13 (36)	14 (46)
40–49	8 (67)	0	5 (17)
>50	1 (8)	1 (3)	5 (17)
Marital Status			
Single	0	8 (22)	3 (10)
Married	10 (83)	28 (78)	27 (90)
Widow	2 (17)	0	0
Education			
None	0	5 (14)	0
Primary	0	17 (47)	1 (3)
Secondary	1 (8)	9 (25)	6 (20)
College	2 (17)	4 (11)	20 (67)
University	9 (75)	1 (3)	3 (10)
Years of Leadership in MCH			
<2	1 (8)	N/A	N/A
2–4	2 (17)	N/A	N/A
>5	9 (75)	N/A	N/A
Number of Children			
1–2	NA	21 (58)	N/A
3–4	N/A	8 (22)	N/A
>5	N/A	7 (20)	N/A

### 3.2. Overview of the findings

Two key themes emerged from this study (i) benefits of good nurse-clients relationships and (ii) disadvantages/negative consequences of poor nurse-client relationships. Key subthemes for the perceived benefits of good nurse-client relationships included: benefits for clients; benefits for nurses; and benefits for the healthcare facility/system. Similarly key subthemes for the disadvantages of poor nurse-client relationships included: disadvantages to nurses; disadvantages to the clients; and disadvantages to the health facility/system. Each of these themes and subthemes is examined in detail below.

### 3.3. Benefits of good nurse-client relationships

The perceived benefits of good nurse-client relationships in MCH services could be heuristically characterized into three groups. The dominant subtheme most participants comprised the benefits for the client seeking MCH care. Almost all participants cited the benefits as including: increased MCH care seeking behaviors, increased willingness and readiness of the client receive MCH care from a nurse, improved emotional well-being (feeling comfortable, happy, and peace of mind) during and after receiving MCH care, improved disclosure tendencies of MCH problems toward nurses, increased chances of receiving correct treatment and medical advice related to MCH, improved adherence to instructions/treatment after receiving MCH care which may contribute to positive MCH health outcomes, reduced costs related to shopping around different MCH clinics for better care, increased client trust of nurses and the healthcare system, increased likelihood of return for MCH care, and increased referral tendencies to MCH clinics. The second group cited by majority of participants encompassed benefits to nurses, including: increased enjoyment and love of nursing and MCH service provision, and increased confidence, efficiency, and freedom of working within MCH services. Moreover, good nurse-client relationships was mentioned by many participants to promote greater interprofessional support among MCH nurses, increased adherence to infection prevention measures to protect patients seeking MCH care, along with increased positivity and the likelihood of receiving support from the community. The third category cited by majority of participants was benefits to the health facility/system, which included: an increased number of MCH clients/referral tendencies (and therefore increased income of the healthcare facility), reduced MCH client complaints and legal actions toward facilities, increased facility delivery, and continuity of MCH care. Overall, good nurse-client relationships was described by almost all participants to contribute to improving MCH outcomes, reducing deaths, and promoting a better image of the health sector (Table 2). Some participants commented:

**Table 2:** Perceived benefits of good nurse-client relationships and disadvantages of poor nurse-client relationships .

Themes	Benefits of good nurse-client relationships	Disadvantages/negative consequences of poor nurse-client relationships
For the client	• Increases healthcare seeking and coming early to a facility (9/9 FGDs;12/12 KIIs). • Increases likelihood of receiving appropriate health education and understanding and adherence to medical advice and instructions (9/9 FGDs;12/12 KIIs). • Increases disclosure tendencies (9/9 FGDs;11/12 KIIs). • Increases likelihood of client satisfaction with care (9/9 FGDs;12/12 KIIs). • Contributes to better maternal and child outcomes (9/9 FGDs;11/12 KIIs). • Increases the chance of the client returning for care (9/9 FGDs;10/12 KIIs). • The client is free to explain medical concerns without fear and without hiding information (8/9 FGDs;11/12 KIIs). • Increases referral tendencies among clients (8/9 FGDs;10/12 KIIs). • Promotes feelings of comfort, happiness, and peace of mind (7/9 FGDs;9/12 KIIs). • Increases willingness to receive care and medical advice offered by nurses (6/9 FGDs;9/12 KIIs). • Client develops trust in the provider and hope of getting well (7/9 FGDs;5/12 KIIs). • Builds peace, friendship, closeness, and tolerance (6/9 FGDs;9/12 KIIs). • Client receives correct and timely care (5/9 FGDs;8/12 KIIs). • Reduces costs to clients from shopping around facilities (5/9 FGDs;6/12 KIIs). • Client feels well before treatment/medications (e.g., reduced pain) (5/9 FGDs;5/12 KIIs). • Increases likelihood of client consent for treatment procedures (5/9 FGDs;5/12 KIIs). • Clients become ambassadors within the community by spreading the word about good nurses (5/9 FGDs;5/12 KIIs)	• Reduces early healthcare seeking behaviors (9/9 FGDs;12/12 KIIs). • Contributes to clients’ mistreatment in healthcare settings (poor reception, bad services, and not receiving correct treatment or the service that one deserves) (9/9 FGDs;12/12 KIIs). • Clients become fearful and lack the confidence to ask anything of the nurse (9/9 FGDs;12/12 KIIs). • Reduces disclosure and limits freedom to explain one's concerns to the nurse leading to an incorrect diagnosis (9/9 FGDs;12/12 KIIs). • Increases client dissatisfaction with care (9/9 FGDs;12/12 KIIs). • Reduces clients’ adherence to nurses’ advice, education, and instructions (9/9 FGDs;12/12 KIIs). • Reduces the likelihood of clients returning for care and increases chances of defaulting care or avoiding the nurse (9/9 FGDs;12/12 KIIs). • Contributes to poor maternal and child outcomes (9/9 FGDs;12/12 KIIs). • May contribute to negative health consequences to the client (e.g., disability, poor health outcomes, or even death) (9/9 FGDs;11/12 KIIs). • Reduces client participation in healthcare decision-making (9/9 FGDs;10/12 KIIs). • Increases the likelihood of clients being verbally and physically abused by nurses (9/9 FGDs;9/12 KIIs). • Increases client distrust of the nurse and treatment received, which may delay healing or chronicity (9/9 FGDs;10/12 KIIs). • Clients cannot receive correct health education and understand instructions (8/9 FGDs;10/12 KIIs). • Increases worries and loss of faith in healthcare (8/9 FGDs;10/12 KIIs). • Increases stress-related diseases and depression (5/9 FGDs;4/12 KIIs)
For the nurse	• Increases desire to go to work every day, job satisfaction, enjoyment, and freedom of working (9/9 FGDs;12/12 KIIs). • Increases confidence, productivity, efficiency, and efforts to offer better care (9/9 FGDs;11/12 KIIs). • Builds client trust toward nurses, which promotes disclosure and adherence (9/9 FGDs;11/12 KIIs). • Increases comfort and peace of mind among nurses (9/9 FGDs;11/12 KIIs). • Increases nurses’ reputation among clients and within communities which fuels referral tendencies and closeness to the community (9/9 FGDs;11/12 KIIs). • Increases love and friendship between nurses and clients (8/9 FGDs;10/12 KIIs). • Increases the number of clients seeking care from the nurse (8/9 FGDs;10/12 KIIs). • Increases cooperation and the likelihood of receiving support from community members when faced with personal problems (8/9 FGDs;7/12 KIIs). • Improves work relationships and teamwork among nurses (6/9 FGDs;6/12 KIIs). • Increases likelihood of adherence to healthcare guidelines and infection prevention and control measures to protect clients (5/9 FGDs;6/12 KIIs). • Increases interprofessional support (i.e., the likelihood of nurses receiving timely support from clients in other professions) (6/9 FGDs;6/12 KIIs). • Increased chance of receiving gifts from clients and community members (6/9 FGDs;5/12 KIIs).	• Reduces work morale and job satisfaction (9/9 FGDs;12/12 KIIs). • Reduces efficiency and productivity (9/9 FGDs;12/12 KIIs). • Reduces friendship, cooperation, and shared decision-making (9/9 FGDs;12/12 KIIs). • Reduces client trust in nurses (9/9 FGDs;12/12 KIIs). • Nurse fails to offer correct management (9/9 FGDs;11/12 KIIs). • Increases client's selectivity of nurses (avoidance of certain nurses); consequently, the nurse loses clients (9/9 FGDs;11/12 KIIs). • Contributes to the negative reputation of a nurse in the community, which may further contribute to segregation and lack of support from community members and neighbours (9/9 FGDs;10/12 KIIs). • Reduces freedom of performing one's duties (7/9 FGDs;8/12 KIIs). • Increases nurses’ retaliation tendencies toward clients (e.g., intentional delay in providing care, which may lead to client death or disability) (6/9 FGDs;7/12 KIIs). • Nurse is portrayed as “bad” and hated by clients, which may increase clients’ complaints about the nurse (consequently loss of job) (5/9 FGDs;6/12 KIIs). • Increases the likelihood of nurses receiving verbal and physical abuse from clients and relatives (4/9 FGDs;5/12 KIIs). • Contributes to further change of behaviours (i.e., a nurse becomes harsh, fails to care for and listen to clients) (4/9 FGDs;4/12 KIIs).
For healthcare facilities and health systems	• Improves the reputation of the facility in the community (9/9 FGDs;12/12 KIIs). • Reduces complaints and conflicts within the healthcare (9/9 FGDs;12/12 KIIs). • Contributes to reducing maternal and child deaths (9/9 FGDs;11/12 KIIs). • Improves service delivery and reduces corruption (9/9 FGDs;10/12 KIIs). • Increases the number of clients (and thereby facility income) (8/9 FGDs;11/12 KIIs). • Reduces clients who go to private facilities where the cost of care is high (8/9 FGDs;10/12 KIIs). • Increases recognition and value of the nursing profession (8/9 FGDs;10/12 KIIs). • Increases community members’ trust toward providers, formal healthcare services, and the health sector (8/9 FGDs;10/12 KIIs). • Promotes correct diagnosis through disclosure; therefore, less admission and short hospital stays (8/9 FGDs;9/12 KIIs). • Increases facility delivery and reduces the number of clients going to traditional healers or home deliveries (8/9 FGDs; 9/12 KIIs). • Health services will improve, and guidelines will be adhered to (4/9 FGDs;3/12 KIIs). • Reduces healthcare budget on medication because of acceptance and implementation of preventive health education offered by nurses (2/9 FGDs;3/12 KIIs).	• Contributes to poor quality of MCH services (9/9 FGDs;12/12 KIIs). • Contributes to the negative reputation of the facility and healthcare sector in communities (9/9 FGDs;12/12 KIIs). • Reduces the number of clients and contributes to loss of income (9/9 FGDs;12/12 KIIs). • Contributes to clients defaulting to other facilities or traditional healers (9/9 FGDs;12/12 KIIs). • Clients lose trust in the facility, formal healthcare, and health sector (9/9 FGDs;12/12 KIIs). • Contributes to loss of resources (e.g., expiry of medications because clients are not coming/returning for care) (9/9 FGDs;11/12 KIIs). • Increases avoidable maternal and child deaths (8/9 FGDs;10/12 KIIs). • Contributes to complaints and complaints and legal disputes toward facilities (5/9 FGDs;6/12 KIIs). • Increases home delivery (5/9 FGDs;4/12 KIIs). • Reduces performance on maternal and child healthcare indicators (3/9 FGDs;4/12 KIIs). • Increases healthcare expenditure because of poor uptake and adherence to preventive measures and education offered by nurses (3/9 FGDs;3/12 KIIs).

*The most significant benefit is feeling well before even one has received treatment because of trust. The patient becomes free to express all their concerns … discloses information to the nurse, and feels valued and cared for by the nurse, which in turn makes her feel well before receiving treatment. (Nurse-midwife, Dispensary)*

*The client and nurses live the same community … they meet in the market, churches, mosques, and other community groups. If there is a good relationship at the hospital, it strengthens the relationships in the community … It makes it easy for the nurse to receive social support within the community. (MCH administrator)*

*When nurses and clients have a good relationship, maternal and child deaths will decrease because patients will be going to the hospital much earlier without fear. We saw many women giving birth at home for fear of the nurses' poor services; consequently, we witnessed many deaths … people will make use of hospital services instead of going to traditional healers. (Client, Hospital)*

### 3.4. Disadvantages of poor nurse-client relationships

The negative consequences of poor nurse-client relationships in MCH care were also mentioned by a majority of participants. From the clients' perspective, most participants linked poor nurse-client relationships to reduced MCHcare seeking tendencies by clients, poor disclosure tendencies among clients and poor adherence to medical interventions and advice. Few participants mentioned poor continuity of MCH care because some clients may prefer home delivery and traditional healers than formal MCH providers. From the nurses' perspective, most participants linked poor nurse-client relationships to reduced work morale, reduced efficiency, reduced productivity, increased likelihood of verbal and physical abuse in MCH care, loss of job due to MCH client complaints and declining trust and reputation. Some mentioned increased abuse of nurses from MCH clients/community members, and lack of support from community members for which MCH services is provided. Finally, from the health facility/system perspective, many participants poor nurse-client relationships linked to declining reputation of the healthcare facilities and MCH clinics and reduced client load in MCH clinics, which may to either underutilization of resources and declining income (particularly in for-profit healthcare institutions). Other important observations cited by some participants were the description that poor nurse-client relationships may increase complaints and conflicts that may fuel legal disputes between MCH clients, nurses and healthcare facilities, and a preference for home delivery and traditional healers over formal care, which may contribute to increased maternal and child deaths due to substandard care. Some participants commented:

*Bad relationship negatively impacts maternal and child health services … There is a particular nurse; if I go to the regional hospital and find her, I avoid receiving care from her altogether. I [am] better [to] go back home without treatment or to a traditional healer… (Client, Health center)*

*…not receiving correct care. Then, you are not free to explain all your problems to the nurse … you don't get an opportunity to learn what you do not know, and you do not get a chance to learn from the nurse about what you need to do or not do. (Client, Dispensary)*

*The poor relationship creates hate … the client leaves with a bad image of the nurse, and she will send wrong information to the community creating a negative reputation of the nurse. The community becomes fearful of coming to the facility for treatment; consequently, women deliver at home, and we end up with deaths… (Nurse, Health center)*

*[Bad relationship] will contribute to the expiry of medications at the facility because people will avoid coming to the facility with bad nurses and prefer to go for traditional herbs. Attendance to the facility will drop, and the facility income will go down … (Nurse, Dispensary)*

*The nurse will be verbally abused and sometimes beaten, creating long-term conflicts. The nurse loses confidence and cannot offer care freely. (MCH administrator)*

The perceived benefits of and disadvantages associated with good and poor nurse-client relationships cited by majority of the participants highlighted the crucial role of good nurse-client relationships in facilitating positive MCH outcomes and satisfaction for clients, nurses, and health facilities.

## 4. Discussion

The findings from this study contribute to improving understanding of the perceived benefits of good nurse-client relationships and disadvantages of poor nurse-client relationships in MCH from the perspectives of nurses, clients, and administrators in low-income rural contexts. This recognizes the value of nurses/midwives in enhancing client uptake and continuity of MCH care as well as the overall quality of MCH care and positive MCH outcomes and practices, such as strengthened breastfeeding practice and reducing unnecessary cesarean sections, maternal infections, postpartum hemorrhage, and preterm births (8–10).

The benefits for clients from good nurse-client relationships that emerged from this study were improving clients’ MCH care seeking behaviors, readiness to receive MCH care from nurses that may result in enhanced engagement in treatment decisions, development of a trusting relationship, and achieving positive MCH outcomes. It is important to note that similar findings have been reported in studies focused on interpersonal relationships in therapeutic care across Asia and some sub-Saharan African countries (10, 21–24). Previous studies emphasized that good interpersonal relationships between healthcare providers and clients could increase clients' tendencies of seeking formal healthcare services and that such tendencies may facilitate positive patient experiences and health outcomes (10, 21–26). This is critical, particularly in rural areas where home deliveries and medical pluralism are common, which may contribute to poor maternal and newborn outcomes (11). This suggests that strengthening nurse-client relationships may be an important tool for promoting the uptake of formal MCH care and facility-based delivery, thereby improving maternal and newborn health outcomes.

A good relationship involves acknowledging individual clients' uniqueness and engaging them with respect as partners in their care (27). This may explain why most participants suggested that good nurse-client relationships could promote trust, disclosure tendencies, and engagement in MCH care among clients. In addition, our findings indicate that among the benefits of good nurse-client relationship is patient involvement and participation in planning of their MCH care. This was perceived to reduce the chance of treatment-related errors because MCH nurses have the opportunity to receive adequate information from clients. These findings were consistent with existing evidence that suggested good provider-client relationships promoted trust and created a positive environment for clients to engage in decision-making, openly express their medical concerns, and offer accurate information (10, 21, 22, 27, 28). Furthermore, such client engagement in MCH care may assist providers in arriving at a correct diagnosis and offering appropriate treatment interventions (1, 5, 14). This suggests that positive nurse-client relationships may create trust in providers among clients within MCH services that may facilitate taking a thorough medical history, which is crucial in diagnosing actual and potential health problems affecting MCH clients and offering timely and appropriate interventions.

Among the important benefits of nurse-client relationships that emerged from this study was that good nurse-client relationship increased clients' adherence to instructions given by MCH nurses/midwives and to their treatment regimens, which contribute to positive MCH health outcomes (physical, emotional, and social-economic health). Furthermore, good relationships were linked to an increased likelihood of returning to the facility for MCH care (continuity of care) and timely referrals. These findings are important because poor adherence to medical advice and discontinuity with care are among the common challenges in MCH care. Consequently, previous studies emphasized the value of strengthening nurse-client relationships to partly address these problems (10, 21, 29–31). The present findings were similar to those in previous studies that found positive provider-client relationships had multiple positive effects, mainly compliance with treatment plans and improved quality of care (15).

Unlike previous studies, participants in this study believed that good nurse-client relationships could be cost-effective by reducing the costs related to the tendency of some clients to “shop around” different MCH clinics/providers when dissatisfied with care in one facility. Good relationships were also linked to a reduced tendency toward medical pluralism or preference for traditional therapies, which facilitated timely access to appropriate formal treatments. Most participants felt that with good relationships, MCH clients would seek healthcare services immediately on feeling unwell or starting labor, and receive skilled and timely care. Consistent with previous studies (32–34), participants in this study related good interpersonal relationships to satisfactory, timely, safe, and quality care, including close monitoring and protection from nosocomial infections, particularly during labor and delivery. Collectively, these accounts suggested that good nurse-client relationships could impact clients' MCH seeking behaviors, disclosure, engagement, and adherence, and consequently improve MCH care outcomes.

Previous studies on nurse-client relationships did not focus on its benefits to the nurses and midwives. This study highlighted the perceived benefits of good nurse-client relationships for MCH nurses/midwives, including generating a positive image and reputation in the communities served, increased peace of mind and happiness, and smooth transactions with clients that increased confidence, efficiency, and job satisfaction. In contrast, previous studies associated nurses' job satisfaction with a conducive work environment and leadership, salary and fringe benefits, and the availability of supplies and equipment for work (17, 21, 35–37) rather than as a result of good interpersonal relationships between nurses and clients. The present findings indicated that with good nurse-client relationships, nurses may enjoy and love their job more, and therefore become more autonomous, confident, and efficient with increased productivity with MCH services. It is natural for human beings to like being appreciated; with good interpersonal relationships, nurses/midwives will earn respect from their community, thereby boosting their morale and self-determination (38). This suggests that strengthening nurse-client relationships may contribute to MCH nurses' job satisfaction, even without attention to other satisfaction variables.

The present study indicated that the benefits of good nurse-client relationships extend beyond nurses and clients to the healthcare system. Good nurse-client relationships were linked to an increased referral tendency among MCH clients which in turn may increase income to the healthcare facility. There were suggestions that good nurse-client relationships may reduce legal conflicts as well as reduce negativity and complaints of MCH clients towards healthcare facilities and the healthcare system. An increase in facility delivery and skilled birth attendants among pregnant women in areas where home deliveries are still prevalent was suggested as a key benefit of good nurse-client relationships which may consequently reduce maternal and child deaths. Similar findings have been widely documented in previous studies on provider-client relationships within the country and beyond (10, 15, 29–36). This suggests that improving nurse-client relationships may be a key strategy for improving MCH indicators more broadly. The present study indicated that poor nurse-client relationships may contribute to reduced MCH care seeking tendencies, insufficient disclosure, poor adherence to treatment regimens, poor continuity of care, and a preference for home delivery and traditional healers. This may be attributed to fear of hostility, poor MCH services by nurses, and lack of awareness of the client's rights by both clients and nurses with MCH service points. These results were similar to previous studies that indicated the consequences of poor nurse-client interpersonal relationships in MCH were disrespectful maternal care and poor mother-baby outcomes, which have been documented worldwide, with context-dependent variations (2, 22, 28, 39). In addition, poor nurse-client relationships reduce satisfaction with the MCH care provided by nurses and clients' involvement in their care, rendering them passive care recipients (15). Furthermore, poor nurse-client relationships have been found to affect women's readiness and willingness to access skilled maternal healthcare in a health facility (22, 28, 29). In most cases, a client will never report or appreciate how technically efficient the nurse was in providing care. However, the interpersonal relationship experienced during the care process underscores how good or bad the care was and influences whether the person will continue seeking care at the facility (40, 41).

In the present study, most participants thought that poor nurse-client relationships would reduce work enjoyment among nurses and mean they would be dissatisfied with their job. Low productivity implies that poor relationships negatively affect staff morale and satisfaction; if not addressed, it further affects productivity and increases costs due to inefficiencies. In contrast to this study, previous studies associated nurses' job dissatisfaction with low salaries and remuneration, poor work environment, and scarcity of work tools and supplies (as noted above). Furthermore, the present findings indicated that nurses/midwives that had poor relationships with their clients were likely to contribute to medical errors (commission or omission), wrong medications, and inconsistent MCH care among nurses. This erratic MCH care could result from inadequate communication and patient involvement in care planning. Implications of erratic and improper care for clients include prolonged hospital stay, nosocomial infections, and other adverse effects, including death (12, 40).

The public image of nurses/midwives depends on their ability to communicate and establish good interpersonal relationships. In poor relationships, irrespective of how technically apt the provider may be, the profession's reputation is tarnished in the eyes of the public and may become unpopular (5, 9, 42). The present findings indicated that poor interpersonal relationships placed nurses/midwives at high risk for verbal and physical abuse from dissatisfied MCH clients. Previous studies highlighted several physical and verbal attacks on nurses and midwives following unsuccessful treatment where they were held responsible for the loss/failure (10, 21, 43). In addition, poor nurse-client relationships reduce clients' trust, which complicates the MCH care delivery process. Evidence has shown a relationship between trust in healthcare providers and increased compliance with the treatment regimen, follow-up care, and use of healthcare services (10, 21, 38). When there is no trusting relationship, the clients will not seek formal health services or return for a follow-up visit, and may not adhere to medical advice, which may increase the likelihood of poor health outcomes.

Findings from this study indicated that the main disadvantages of poor nurse-client relationships to the healthcare system were poor quality of services and an increased burden of healthcare costs. MCH clients may be mismanaged, which may contribute to legal actions and complaints along with issues such as misdiagnoses and prolonged hospital stay (1, 7, 10, 29, 44). Loss of clients due to underutilization of health services may also reduce the facility's performance on key MCH indicators.

### 4.1. Limitations

This study had two main limitations. First, this study was conducted as part of a larger study that examined factors that shaped nurse-client relationships with the aim of co-designing acceptable interventions. Therefore, the list of benefits/disadvantages associated with good and poor nurse-client relationships presented may not be exhaustive. Furthermore, this study used nurses as an exemplar of providers to examine nurse-client relationships in MCH in a rural setting. However, patients interact with a multidisciplinary team of providers in healthcare settings, meaning their perceptions of benefits and disadvantages may reflect such interactions. Conducting a similar study with other providers and in different settings may yield different benefits and disadvantages. However, as this was the first such study in this context, further inquiries may extend beyond the nursing profession and rural contexts.

## 5. Conclusion

The nurse-client relationship is an essential aspect of MCH care that can benefit patients, nurses, and the healthcare system. Therefore, identifying and implementing feasible and acceptable interventions for nurses and clients to pave the way for good nurse-client relationships may improve MCH outcomes and performance indicators.

## Data Availability

The original contributions presented in the study are included in the article/**Supplementary Material**, further inquiries can be directed to the corresponding author.
