# Supplementary material for: The role of nurse-client relationships in maternal and child healthcare: a qualitative study in rural Tanzania
Source: Front Health Serv. 2023 Jun 26;3:1058840. doi: 10.3389/frhs.2023.1058840 (PMC10331615; doi:10.3389/frhs.2023.1058840)
Supplement: Supplementary file 1 [file Datasheet1.docx]

***Supplemental Material***

***Interview Guide***

**PART 1: FOCUS GROUP DISCUSSION WITH NURSES AND CLIENTS**

- *Ensure availability of a quiet and safe venue*
- *Welcome all the participants.*
- *Read the consent form.*

**1: PARTICIPANTS' DEMOGRAPHIC INFORMATION**

| PARTICIPANT | AGE | GENDER | MARITAL STATUS | LEVEL OF LICENCE  (NURSES ONLY) | HIGHEST LEVEL OF EDUCATION | LEVEL OF HEALTH FACILITY | YEARS OF MCH CARE EXPERIENCE |
| --- | --- | --- | --- | --- | --- | --- | --- |
| 1 |  |  |  |  |  |  |  |
| 2 |  |  |  |  |  |  |  |
| 3 |  |  |  |  |  |  |  |
| 4 |  |  |  |  |  |  |  |
| 5 |  |  |  |  |  |  |  |
| 6 |  |  |  |  |  |  |  |
| 7 |  |  |  |  |  |  |  |
| 8 |  |  |  |  |  |  |  |

Facilitator______________________

Notetaker______________________

Starting time___________________

**2: FGD QUESTIONS**

1. What does a good nurse-client relationship mean to you? (*Probe: ever experienced good relationship with nurse/clients? What happened?)*
2. What does a bad/poor nurse-client relationship mean to you? (*Probe: ever experienced bad relationship with nurse/clients? What happened?)*
3. What are the benefits of having a good relationship with your nurse/client in MCH care?*(Probe: benefits to the client, benefits to the nurse and benefits to the healthcare facility/system?)*
4. What are the consequences/disadvantages of having a bad relationship with your nurse/client in MCH care? *(Probe:* *consequence to nurses, consequence to clients, consequence to health system?*
5. What are the contributors to a bad relationship between nurses and their clients in MCH care in Shinyanga? *(Probe: Nurse factors, client factors, health system factors?)*
6. What are the existing strategies for strengthening nurse-client relationships in MCH care in Shinyanga?
7. Any other comment in relation to the nurse-client relationship in Shinyanga?

THANK YOU End Time_________________________

**PART 2: KII WITH MCH ADMINISTRATORS**

- *Ensure availability of a quiet and safe venue*
- *Welcome the participant.*
- *Read the consent form.*

Interviewer ______________________

Starting time_____________________

**1: PARTICIPANT DEMOGRAPHIC INFORMATION**

1. Participant title: ________________________
2. Age_____________________________________
3. Gender_________________________________
4. Highest level of Education_____________
5. Marital Status___________________________
6. Years of MCH leadership________________

**2: KII QUESTIONS**

1. What are your roles and responsibilities in relation to MCH care?
2. What does a good nurse-client relationship mean to you? (*Probe: ever received clients compliments of nurses? What happened?)*
3. What does a bad/poor nurse-client relationship mean to you? (*Probe: ever handled client’s complaints about nurses? What happened?)*
4. What are the benefits of having a good relationship between a nurse and client in MCH care? *(Probe: benefits to the client, benefits to the nurse and benefits to the healthcare facility/system?)*
5. How a poof relationship between nurses and clients have impacted MCH care in Shinyanga? *(Probe: consequence to nurses, consequence to clients, consequence to the health system?*
6. What are the contributors to a bad relationship between nurses and their clients in MCH care in Shinyanga? *(Probe: Nurse factors, client factors, health system factors?)*
7. What are the existing strategies employed for strengthening nurse-client relationships in MCH care in Shinyanga?
8. Any other comment in relation to the nurse-client relationship in Shinyanga?

THANK YOU End Time,_________________________
